# Complication rare du clou gamma: migration de vis cervicale au contact des vaisseaux iliaques

**DOI:** 10.11604/pamj.2014.18.188.4877

**Published:** 2014-07-04

**Authors:** Younes Ouchrif, Issam Elouakili

**Affiliations:** 1 Service de Chirurgie Orthopédique, CHU de Rabat, Maroc

**Keywords:** Clou gamma, vaisseaux iliaques, vis cervicale, gamma nail, iliac vessels, cervical screw

## Image en medicine

La plupart des fractures per et sous trochantériennes consolident après une ostéosynthèse par clou gamma. Néanmoins, dans certaines situations notamment chez les sujets âgées avec une qualité osseuse médiocre un démontage du matériel peut survenir. Celui-ci est suspecté devant une recrudescence de la douleur et l'impotence fonctionnelle totale du membre inférieur. La confirmation diagnostic se fait après radiographie standard qui montre chez notre patient une migration de la vis cervicale en intra pelvien. Malgré un examen vasculo nerveux normal, nous avons réalisé un angioscanner qui a montré le rapport intime de la vis cervicale avec les vaisseaux iliaques. Il faut toujours devant ce tableau éliminer un problème infectieux sous-jacent qui peut être la source du démontage. L'indication thérapeutique diffère en fonction de l’âge du patient. En effet, si chez le sujet jeune le but est d'obtenir une consolidation et d’éviter au maximum le recours à la prothèse totale de la hanche, chez le sujet âgé la déambulation et la marche rapide sont l'objectif principal afin d’éviter chez lui les complications de décubitus souvent mortels, ce qui justifie le recours à des PTH. Chez notre patient la difficulté était la proximité de la vis cervicale des vaisseaux iliaques, la présence d'un chirurgien vasculaire était nécessaire au moment de la reprise.

**Figure 1:**
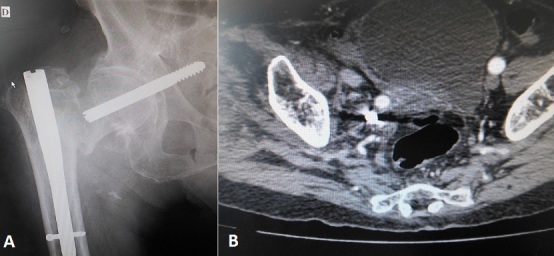
A) radiographie du bassin de face montrant la migration de la vis cervicale en intra pelvien; B) angioscanner pelvien montrant la proximité de la tête de vis des vaisseaux iliaques

